# Supplementary material for: A Flexible and Highly Sensitive Pressure Sensor Based on AgNWs/NRLF for Hand Motion Monitoring
Source: Nanomaterials (Basel). 2019 Jun 29;9(7):945. doi: 10.3390/nano9070945 (PMC6669722; doi:10.3390/nano9070945)
Supplement: Supplementary file 1 [file nanomaterials-09-00945-s001.pdf]

Supplementary Material

A Flexible and Highly Sensitive Pressure Sensor Based on AgNWs/NRLF for Hand Motion Monitoring

Yi Sun [1] and Zhaoqun Du [1,2,*]

[1] Key Laboratory of Textile Science & Technology (Donghua University), Ministry of Education, College of Textiles, Donghua University, Shanghai 201620, China; 2150082@mail.dhu.edu.cn

[2] Jiangxi Provincial Center for Quality Inspection and Supervision on Down Products, Gongqingcheng 332020, China

* Correspondence: duzq@dhu.edu.cn

1. Condition of synthetic process

Figure S1a,b showed that AgNWs were synthesized by magnetic stirring at 150 rpm and 300 rpm for 2 h respectively. As can be seen from Figure S1a, the nanowires were unevenly distributed with few particles, and the average length and diameter of the AgNWs synthesized at 150 rpm were 6 ± 3 μm and 220 ± 14 nm. From Figure S1b, it found that the nanowires were sparsely distributed with less yield and many particles, and some of the nanowires were not in contact with each other. The average length and diameter of the AgNWs synthesized at 300 rpm were 5 ± 2 μm and 174 ± 11 nm. The results found the lower stirring rate improves uniform the diameter and length, and the stirring rate should not be high, nanowires will become short. The appropriate stirring rate can homogenize and control the length and diameter of the synthesized nanowires.

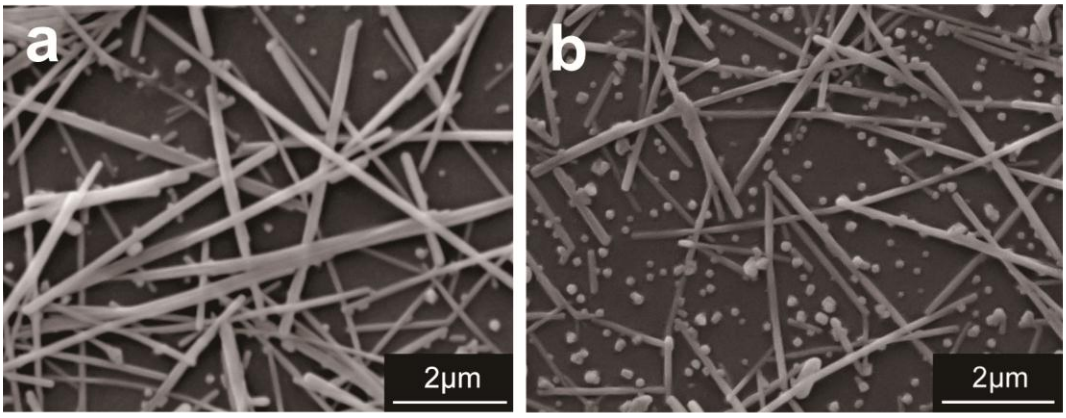

Figure S1. SEM micrographs of various parts of the samples: (**a**) AgNWs synthesized at 150 rpm. (**b**) AgNWs synthesized at 300 rpm.

2. Performance of dip-coated AgNWs-NRLFs

In Figure S2a,b, the contact angles of the 6th and 7th dip-coated samples were 105° and 83° which represent the weakened hydrophobicity and tend to hydrophily. Figure S2c showed the original resistance of the 3th dip-coated were tested as 557 Ω. Figure S2d showed that the piezoresistive effect of 3th dip-coated AgNWs-NRLF when it under pressure and its resistance greatly decreased to 92 Ω when touched by finger. The results demonstrated that the sample could have evident piezoresistive effect after dip-coating and could be applied as pressure sensors.

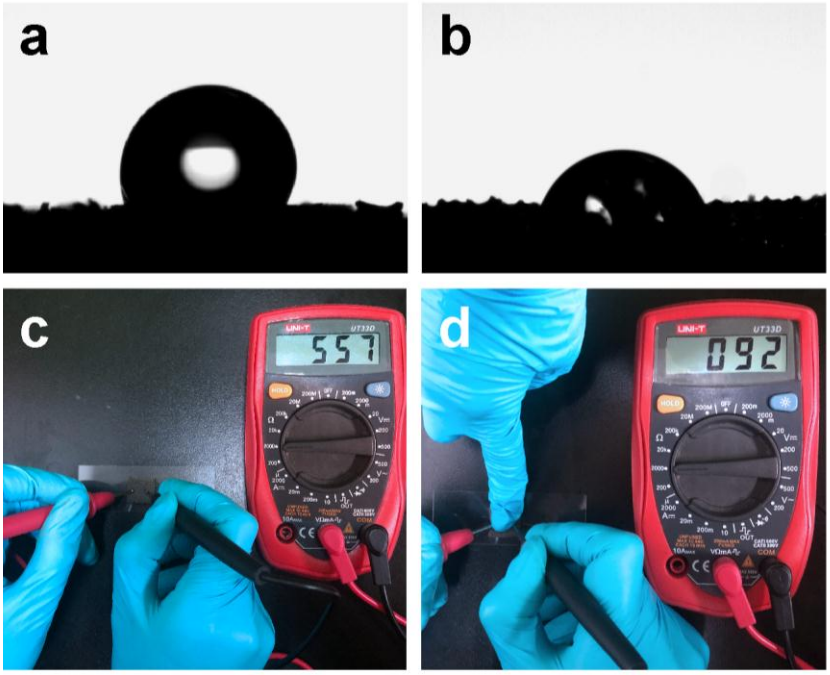

Figure S2. (**a**,**b**) contact angles of 6th and 7th dip-coating cycles. (**c**,**d**) piezoresistive effect of 3th dip-coating AgNWs-NRLF before and after touching by finger.
